# Rare Clinical Presentation of Postmenopausal Endometriosis: A New Perspective

**DOI:** 10.14336/AD.2023.1022

**Published:** 2023-11-05

**Authors:** Natalia Rzewuska, Michał Kunicki, Sylvia Mechsner, Pawel Kordowitzki

**Affiliations:** ^1^Department of Gynecological Endocrinology, Medical University of Warsaw, 00-315 Warsaw, Poland.; ^2^INVICTA Fertility and Reproductive Center, 00-019 Warsaw, Poland.; ^3^Charité-Universitätsmedizin Berlin, Corporate Member of Freie Universität Berlin and Humboldt-Universität zu Berlin, Endometriosis Centre Charité, Department of Gynaecology, 13353 Berlin, Germany.; ^4^Department of Basic and Preclinical Sciences, Faculty of Biological and Veterinary Sciences, Nicolaus Copernicus University, 87-100 Torun, Poland

**Keywords:** menopause, endometriosis, SIRT1, deep infiltrating endometriosis

## Abstract

Endometriosis affects 2-5 % of postmenopausal women with menopause hormone therapy and is less common in women without treatment with exogenous estrogen or tamoxifen. Postmenopausal endometriosis has more unknown aspects in its pathogenesis and clinical manifestation than in the case of premenopausal patients. The aim of this review was to summarize the clinical presentation of rare cases of endometriosis, including deep infiltrating (DIE) and extragenital endometriosis, in women. The symptoms of endometriosis in the post-reproductive age are more heterogeneous than in women of childbearing age, often resembling symptoms of gastrointestinal tumors or urinary tract diseases. We summarize cases of endometriosis of the intestines, liver, pancreas, and stomach, as well as endometriosis of the urinary tract and skin, with non-gynecological manifestations. We also describe the pathogenesis of endometrial tissue activity in the context of reduced estrogen levels after menopause, which is also not clear, and demands more molecular and genetic studies. NAD+-dependent deacetylases called Sirtuins are metabolic sensors for maintaining body homeostasis. In the context of endometriosis, Sirtuins are being studied for their potential role in modulating inflammation, cell proliferation, and sex hormone sensitivity, but their role in postmenopausal endometriosis is not well researched. Treatment in postmenopausal women includes mostly for now surgery, depending on the location of the lesion, and aromatase inhibitors. The complete genetic and epigenetic profile in women post-reproductive age is needed to propose target therapy, especially in severe cases such as endometriosis that is deeply infiltrating and located outside the pelvis.

Clinical presentations of endometriosis in postmenopausal age are heterogeneous in regard to clinical manifestations and patient characteristics [1]. Endometriosis is a common disease, affecting approximately 2 - 10 % of women of childbearing age [2 - 4]. About 190 million women around the world worldwide suffer from this condition [5]. The highest incidence is observed in women between 25 and 45 years of age, with the majority of cases occurring between the ages of 30 and 40. The ovaries, uterosacral ligaments, and peritoneum of the pelvis are the primary and most common locations where endometrial implants are found. Deep infiltrating lesions could develop in the recto vaginal septum (with or without bowel/vaginal infiltration) [1]. After menopause, its incidence is estimated at 2 - 5 %, mainly in women on hormonal replacement therapy (HRT). The risk is increased also in the case of estrogen monotherapy (ERT) and the tamoxifen treatment, as well as in cases of estrogen exposure such as obesity, whereas rarely in women without HRT [5, 6]. ESHRE guideline allows for consideration of combined menopausal hormone therapy for the menopause symptoms in women with a history of endometriosis, but estrogen replacement therapy (ERT) should be avoided. ERT is associated with an increased risk of malignancy [3]. Endometriosis can be characterized as endometrial-like tissue outside the endometrium and myometrium, as a result of retrograde flow of blood and menstrual tissue [3, 7, 8]. This condition is dependent on estrogen and causes clinical manifestations such as pelvic pain, non-menstrual pain, and infertility [1], which seriously affect the quality of women’s lives. As the level of estrogen decreases after menopause in most cases, one could expect relief from the before-mentioned symptoms. Based on clinical, histopathological, and immune-cytochemical differences, one could distinguish the following main types of endometriosis: peritoneal, ovarian (endometriomas), and deep endometriosis, as well as endometriosis occurring in other, distant locations, such as abdominal wall incisions, perineal incisions, and even cases where it invades the pleura (lining of the lungs). The type of deep or ovarian endometriosis is rare in postmenopausal women [8].

Due to the fact that endometriosis has serious implications in women, the aim of this review is to synthesize information regarding rare types of endometriosis in postmenopausal women and gather clinical practical instructions for clinicians.

## Methods

We present a perspective for students and practitioners and, therefore, cited references were chosen on the basis of pre-selected topic areas: pathogenesis of endometriosis in women on HRT, or without HRT therapy, molecular and genetic basis of postmenopausal endometriosis, clinical manifestation of deep infiltrating endometriosis and extragenital endometriosis, as well therapeutic options.

### Pathogenic theories of endometriosis in post-menopausal women

Postmenopausal endometriosis is connected with increased activity of endogenous or exogenous sources of estrogen [9]. In certain instances, it could be explained as a continuation or extension of preexisting endometriosis [10]. HRT was considered as a factor that might reactivate existing endometriosis or lead to the development of new endometrial implants in menopausal women with a history of endometriosis. Nevertheless, it is important to note that endometriosis can also occur in postmenopausal women without HRT [11]. The pathogenesis of this condition is heterogeneous like the disease itself, and it remains elusive. The theory that endometriosis consists of endometrial-like tissue is incorrect, especially in aspects of deep and cystic endometriosis [8]. Multiple different factors may affect the location and clinical course of endometriosis (Table 1), like genetic, epigenetic, immunological, hormonal, and environmental factors. The endometrial cell undergoes a series of genetic or epigenetic transformations, and therefore, occurs in both neonatal and mature adult endometrium. The distinct interplay of genetic and epigenetic events will dictate whether endometriosis develops into its typical, cystic, or deep forms. Moreover, the pathogenesis of active endometriosis after menopause includes some possible mechanisms like adipositas because overweight women have a higher incidence of active endometriosis. The possible source of estrogen is also adrenal and skin, as well as the increased expression of P450 aromatize in ectopic endometrium [12].

### Role of SIRT1 in the pathogenesis of endometriosis

Endometriosis is characterized as an inflammatory condition marked by cell proliferation and resistance to progesterone (P4). Estrogen (E2) plays a dominant role in this process, supporting the survival of ectopic tissue outside the uterus. Reports are suggesting that oxidative stress in cells may be one of the factors in the pathogenesis of endometriosis. The family of NAD+-dependent deacetylases, called Sirtuins, are metabolic sensors for maintaining body homeostasis. Sirtuins (mammal homologs of yeast Sir2) have been discovered and grouped into a family of seven members called class III histone deacetylases (SIRT1-SIRT7). Interestingly, SIRT1 shows the highest phylogenetic similarity to yeast Sir2 and has become the most extensively studied member of this family. This gene was initially considered as being overexpressed in women with endometriosis and affects the resistance to progesterone P4 [13, 14]. Sirtuins collectively respond to various metabolic disruptions, inflammatory cues, as well as hypoxic or oxidative stress, and they play a role in processes related to aging and longevity. Sirtuin 1 activity serves as a protective mechanism against the harmful impacts on fertility caused by conditions including polycystic ovary syndrome (PCOS), endometriosis, diabetes, xenobiotic stress, and even the aging process. SIRT1 plays an important role in the regulation of inflammatory cytokines, which was proved by experiments conducted with primary endometriotic stromal cells and peritoneal immune cells. Sirtuin activators have a potential role as inhibitors of the inflammatory response in endometrial lesions. In consequence, SIRT1 appears to play a possible role as a target for new therapeutic strategies in endometriosis [13].

### Manifestation of the postmenopausal endometriosis

**Figure 1.**
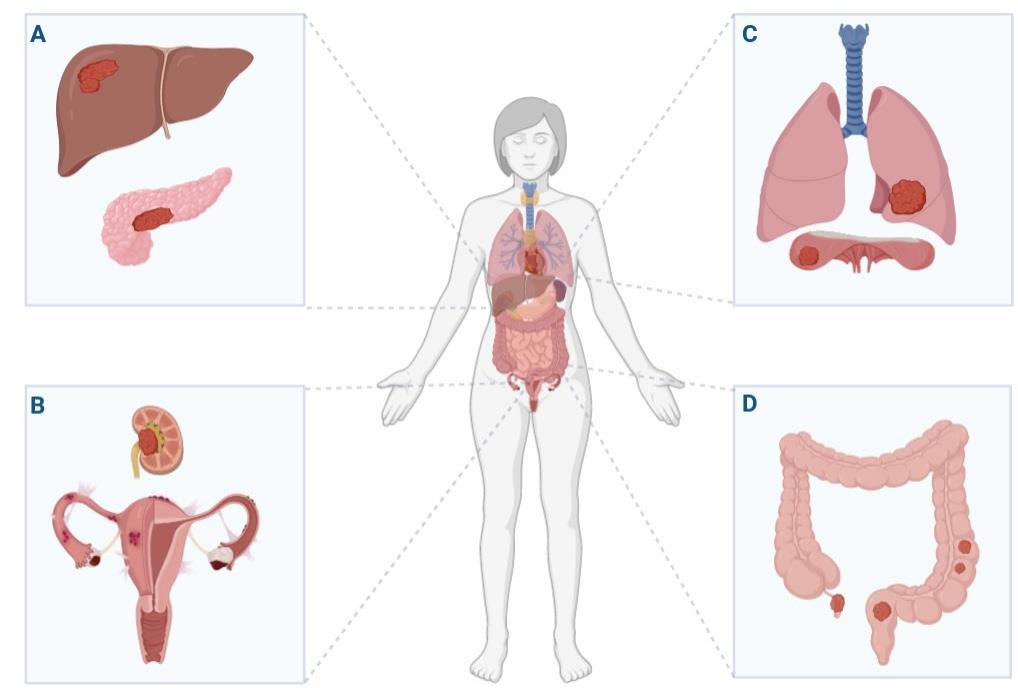
**The localization of extragenital endometriosis in women of postmenopausal age. (A)** Lesions in the liver and pancreas, **(B)** lesions in the urogenital tract, **(C)** lesions in the lungs and diaphragm, **(D)** lesions in the intestine.

Clinical symptoms of postmenopausal endometriosis are heterogeneous and depend on the location of the endometrial tissue lesions (Fig. 1). The main symptoms include pelvic pain, urinary tract symptoms, and narrowing of the small intestine. In the case of deep infiltrating endometriosis (DIE), the lesions that mimic a colon tumor and even the sigmoid colon or cause abdominal hemorrhage have been described [8, 15 - 26]. The other symptoms of DIE include renal failure, hydronephrosis, and urinary bleeding [17, 21, 27 - 28]. An infrequent presentation of endometriosis has also been featured, like vaginal endometrial cysts and vulvar skin lesions [8, 25, 29, 30]. Due to the diverse clinical manifestations, endometriosis may resemble a neoplastic lesion. It could mimic a stomach or pancreatic tumor, as it was presented in the literature [26, 28]. It remains unclear why postmenopausal women who do not receive MHT may continue to experience disease progression even ten years after menopause [8].

**Table 1:** Selected examples of location and clinical manifestation of deep infiltrating endometriosis and extragenital endometriosis in postmenopausal women.

Study (Author, year)	Locations of the lesions	Symptoms & sign
**de Almeida Asencio, F (2019)** [8]	retro-cervical and right pararectal localization	pelvic pain
**de Almeida Asencio, F (2019)** [8]	pouch of Douglas, rectum	pelvic pain, dyspareunia, dyschezia, and repetitive constipation
**de Almeida Asencio, F (2019)** [8]	cecum	severe pain
**de Almeida Asencio, F (2019)** [8]	retrocervical and sigmoid colon	increasing severe pelvic pain (10/10) and rectal pain with tenesmus
**de Almeida Asencio, F (2019)** [8]	pouch of Douglas, right ureter (deep endometriosis nodule)	severe pain
**de Almeida Asencio, F (2019)** [8]	retro-para-cervical endometriosis	chronic pelvic pain, dyschezia, dyspareunia intermittent diarrhea
**Djursing H (1981)** [9]	diffuse pelvic endometriosis and intestinal	vaginal bleeding and increasing abdominal pain
**Yukumi, S. (2020)** [16]	thoracic endometriosis	recurrent left pneumothorax
**Bese T (2003)** [19]	extensive pelvic endometriosis (rectal muscular wall endometriosis)	uterine bleeding
**Snyder BM. (2018)** [1]	transverse colon	iron deficiency anemia; Blood in the stool
**Deval B (2002)** [15]	colon endometrioma	pelvic pain, vaginal discharge, constipation, and a weight loss of 30 kg progressing over a 6-month period
**Goldsmith, P. J. (2009)** [18]	liver	right upper quadrant pain and a cystic liver mass
**Verbeke, C (1996)** [35]	liver	right epigastric pain
**Abdallah A.A (2016)** [26]	gastric endometriosis	epigastric pain, left loin pain, nausea
**Izuishi K (2015)** [20]	small bowel	severe abdominal pain and vomiting
**Klenov VE (2015)** [21]	invasive pelvic endometriosis	vaginal bleeding, hematuria
**Petros JG (1992)** [22]	colonic endometriosis (two cases)	colonic obstruction
**Kempers (1960)** [23]	intestinal (8 cases)	pain
**Popoutchi P (2008)** [24]	sigmoid, rectum	hematochezia and tenesmus, pelvic pain, liquid feces, pelvic pain
**Choi JK (2020)** [25]	primary cutaneous endometriosis of the navel	bump in the navel, umbilical pain
**Abdallah A.A (2016)** [26]	gastric endometriosis	epigastric pain, left loin pain, nausea
**al-Izzi MS (1989)** [27]	bladder wall	hematuria
**Plodeck V (2016)** [28]	pancreas	left upper quadrant pain
**Sarpietro (2022)** [29]	vulva	vulvar lump
**Corazza M** [30]	vulva	blackish macule and a small central ulcer

### Management and diagnosis

Postmenopausal endometriosis rarely occurs with specific symptomatology, leading to frequent misdiagnoses with other conditions. Besides gynecological symptoms, postmenopausal endometriosis can manifest with other symptoms involving the intestines, urinary system, and in some cases, less common symptoms depend on its location [8]. The ESHRE “Guideline for the Diagnosis and Treatment of Endometriosis (2005)” and the ESHRE “Guideline: Management of Women with Endometriosis (2013)” are previous management of endometriosis in medical practice [31, 32]. Currently, the standard of the treatment designates ESHRE guideline: endometriosis from 2022 [3]. However, the clinical management of postmenopausal endometriosis is an insufficiently described issue and the usage of combined HRT remains unclear [33]. The diagnosis is based on imaging techniques, which are mainly used in clinical practice, including transvaginal sonography (TVS) and Magnetic resonance imaging (MRI), computed tomography (CT), rectal endoscopic sonography, and three-dimensional (3D) ultrasound [5, 34]. While these tools are important imaging investigations, they can be more challenging to interpret in menopausal patients compared to younger patients because of the higher likelihood of neoplastic lesions and the diverse appearance of endometriosis in menopausal patients [5]. MRI and transvaginal ultrasonography have an implementation in types of deep endometriosis but are worst applied in peritoneal endometriosis. Research conducted in recent years has revealed that the dysregulation of microRNA (miRNA) holds diagnostic significance and can be detected in a blood sample [34]. Recently, the gold standard for diagnosing endometriosis, regardless of age, involves performing laparoscopy and biopsy with histopathological evaluation to confirm suspicious lesions. Until the 2022 guidelines, laparoscopy is the preferred method for examining the pelvis, allowing both diagnosis and treatment of lesions simultaneously [3, 5]. Laparoscopy with a histopathological diagnosis of the lesion is recommended when the results of imaging tests are negative or empirical treatment does not bring successful outcomes [3]. Until the 2022 guidelines, the gold standard in the diagnosis of endometriosis was laparoscopy, which allows for the simultaneous diagnosis and treatment of lesions. Currently, laparoscopy with histopathological diagnosis of the lesion is recommended when the results of imaging tests are negative or empirical treatment does not bring successful results [3, 5].

### Deep infiltrating endometriosis

**Figure 2.**
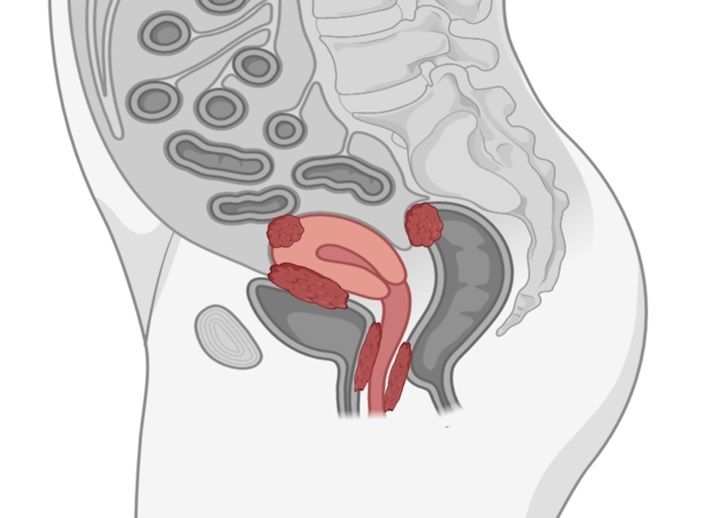
Locations of extrapelvic, deep-infiltrating endometriosis (DIE) in women.

Deep infiltrating endometriosis (DIE) is characterized by endometrial tissue that extends beyond 5 mm beneath the peritoneal surface, often infiltrating organs such as a ureter, bladder, and intestine (Fig. 2). Its prevalence is estimated as 1-2 % of endometriosis. DIE is uncommon and the most difficult type of endometriosis to diagnose and treat when compared to more common ovarian endometriomas and superficial peritoneal endometriosis [35]. Often DIE requires more comprehensive management. In some instances, medical therapy alone may prove effective in alleviating symptoms and addressing the clinical manifestations associated with DIE. Although endometriosis is considered an estrogen-related disorder, the progression in symptoms after menopause in women without MHT is surprising, especially in the case of women without a typical disease history.

### Malignant transformation

Postmenopausal age is the risk factor for malignancy of endometrial lesions; therefore, the assessment of potential malignancy should be conducted in women with postmenopausal endometriosis [1]. The risk of malignancy is estimated as 1 % both for endometrioma and DIE and that is why the Society of Radiologist Ultrasound recommends a minimum of once a year a pelvic ultrasound to monitor the endometriotic lesions, and MRI for DIE should be performed every 6 months and then depends on results one in 2 or 3 years [2]. Malignant transformation may involve both endometriosis-related ovarian cancer (with symptoms of increased abdominal circumference, early feelings of satiety, flatulence, and abdominal pressure) and deeply infiltrating endometriosis (DIE) (described endometrial malignancy in the intestine may present with obstruction or bleeding) [6]. DIE can undergo malignant transformation at an average age of 49, usually to adenocarcinoma. The lesions are usually misdiagnosed as malignant tumors of the rectum or cervix. The DIE exhibits growth into the deeper layers of the peritoneum, promoting the proliferation of deep fibrous connective tissue and the formation of nodules in various organs like the uterus, ureter, vagina, and rectum. Although DIE can locally invade surrounding structures, it rarely metastasizes. The pathogenesis of the malignant transformation of endometrial lesions is not well described, but the immune response and hormonal disorders are taken into account [16]. Reactive oxygen species (ROS) are significant contributors to the development of endometriosis as they increase the levels of free heme and catalytic iron, possibly serving as a fundamental mechanism in the development of endometriosis-associated cancers. Certain specific microRNAs (miRNAs) play a crucial role in tissue repair, also responsible for transforming growth factors, cell growth, proliferation, apoptosis, and angiogenesis. For this reason, miRNAs can be associated with the malignant progression of endometriosis [1, 34].

### Active extragonadal endometriosis in postmenopausal women

The literature indicates distant and extragenital locations of endometriosis, which are extremely rare findings after menopause. Extragonadal location is better described in premenopausal women. The most common locations of extrapelvic endometriosis include gastrointestinal, urinary, and retroperitoneal lesions, as well as abdominal wall and even thoracic location [28, 35, 36]. The frequency of endometriosis affecting the intestines varies between 3% to 34%. The most commonly affected are the rectum, sigmoid colon, appendix, terminal ileum, and cecum, but there are known cases of localization of endometriosis in the liver in postmenopausal women. In the case of intestinal endometriosis, the main symptoms include pelvic and abdominal pain, dysmenorrhea and dyspareunia, intestinal syndromes like constipation, and 20% rectal bleeding [16, 24, 27, 32]. The pathogenesis of intestinal endometriosis after menopause remains elusive. Diagnosing bowel endometriosis can be challenging, even with colonoscopy, as most cases do not infiltrate beyond the serosa, and only number, extend into the mucosal layer. The challenge in achieving complete elimination of bowel endometriosis and achieving long-term symptom reduction arises from the strong association of endometriosis tissue with the enteric nervous system. This connection is also responsible for the array of symptoms observed in deep infiltrating endometriosis of the bowel [24]. The treatment of endometriosis has primarily centered around relieving symptoms and restoring fertility in women of reproductive age, in the case of intensive intestinal infiltration, surgery is the treatment of choice. The case of intestinal obstruction requiring laparotomy has been described [1].

Thoracic endometriosis, known as thoracic endometriosis syndrome (TERP) is considered a rare form of endometriosis, and its occurrence, natural progression, and the effectiveness of treatments for this condition remain largely unknown and not fully understood. The mechanism by which endometrial tissue reaches or originates in the thoracic cavity remains unclear. Several proposed mechanisms include lymphatic or hematogenous embolization from the uterus, retrograde menstruation, and coelomic metaplasia. However, none of these theories provides a definitive explanation for this phenomenon. To diagnose TERP, an intra-operative visual inspection and appropriate histological examination are required [16]. Cutaneous endometriosis is an uncommon but possible location for this disorder and affects less than 5.5% of endometriosis cases. It frequently occurs as a secondary condition following skin trauma, such as after a Caesarean Cesarean section or myomectomy, where endometrial tissue implants on scar tissue in the skin, but there are cases of endometriosis in postmenopausal women without a previous history of surgery [25]. Endometriosis of the perineum and vulva has been documented in the literature, with the most prevalent location being the episiotomy scar, but the primary locations are also known in the literature [27, 29, 30]. The definitive treatment of cutaneous cases is surgical excision [25].

### Treatment of postmenopausal endometriosis

The preferred initial treatment for symptomatic postmenopausal endometriosis is to discontinue menopausal hormone therapy, increase the ratio of progestin to estrogen, or even exclude the estrogen component when no malignancy is suspected. Women receiving tamoxifen therapy should be considered for treatment with aromatase inhibitors. Surgical treatment is recommended if conservative treatment is unsuccessful and if there is a concern for potential malignancy. However, performing surgery on these patients may involve certain risks because of the elderly age, who might have existing medical conditions, and because of increased operative risks [10, 37]. Nevertheless, despite surgical intervention, there remains a possibility of disease recurrence, mainly in cases of bowel endometriosis [25]. As previously mentioned, surgical intervention for deep infiltrating endometriosis in a postmenopausal population is complicated by various factors. Therefore, a more patient-oriented approach considers individual patient factors, such as in this case, where a less invasive method is demonstrated - ablation followed by continuous combined conjugated estrogen/bazedoxifene therapy (CE/BZA). Bazedoxifene is a selective estrogen receptor modulator (SERM) originally intended for osteoporosis treatment for menopausal women [1]. In conservative treatment, aromatase inhibitors are used to decrease estradiol locally produced within the endometriotic tissue. While aromatase inhibitors (AI) are not officially sanctioned for treating endometriosis-associated pain, they can be deemed a viable oral treatment for managing symptomatic postmenopausal endometriosis [2].

Based on the available data, letrozole and anastrozole have shown promising results in reducing both the symptoms and the size of endometriotic lesions. However, postmenopausal women undergoing AI therapy for endometriosis may experience side effects, including hot flushes, vaginal dryness, and arthralgias. The most significant concern with this treatment is the risk of osteoporosis and possible fractures increasing in age in long-term use of AI [1, 2, 38]. Despite various therapeutic approaches, definitive curative therapy for endometriosis remains elusive, and a better understanding of the underlying disease is also required because it can undergo malignant transformation. There is still a lack of molecular factors that would allow us to understand the nature of DIE and indicate possible therapeutic points, especially in relation to endometriosis progressing to malignancy [1, 33].

**Table 2:** An overview of the advantages and disadvantages associated with various treatment methods for postmenopausal endometriosis.

Method	Advantages	Disadvantages
**Aromatase inhibitors (AI)**	reduce symptoms and size of endometriotic lesions [1, 2]	not officially approved for the treatment of pain associated with endometriosis [2]; side effects of such a therapy: hot flushes, vaginal dryness, and arthralgias; osteoporosis and possible fractures increasing in age [1, 2, 38]
**estrogen/bazedoxifene therapy (CE/BZA)**	protective effect of breast and endometrial tissue; prevention of osteoporosis, preventing vasomotor symptoms of menopause acceptable cardiovascular safety profile [1, 39]	increased risk of venous thromboembolism, Muscle spasms, gastrointestinal disorders, oropharyngeal pain, neck pain, dizziness [39]
**Surgical treatment**	allow for histopathological assessment of the lesion in case of its malignancy [25]	increased operative risks in elderly age; possibility of disease recurrence [10, 37]

### Conclusions

Endometriosis, as a systemic disease, requires special attention in postmenopausal women. The clinical picture may vary significantly depending on the specific location. Endometriosis in postmenopausal age may mimic diseases of the different organs, including urinary tract diseases and gastrointestinal cancer. Therefore, clinicians should be aware that possible symptoms of endometriosis originate outside the reproductive organs. Understanding the mechanisms of endometriosis, especially in rare cases of deep infiltrating endometriosis and endometriosis affecting distant organs in postmenopausal age, is still lacking. Molecular and genetic research is necessary to elucidate the transformation of endometriosis into malignancy or the occurrence of endometriosis many years after menopause without exogenous hormonal therapy, such as involving Sirtuins.
